# Tailoring a complex intervention to reduce antidepressants in institutionalized older persons with dementia

**DOI:** 10.1186/s12913-022-08961-9

**Published:** 2022-12-26

**Authors:** Pernille Hølmkjær, Charlotte Vermehren, Anne Holm, Maarten Pieter Rozing, Kirsten Høj, Gritt Overbeck

**Affiliations:** 1 Department of Public Health, Section of General Practice and Research Unit for General Practice, University of Copenhagen, Copenhagen, Denmark; 2 Department of Clinical Pharmacology, University Hospital Copenhagen, Capital Region, Copenhagen, Denmark; 3 Department of Drug Design and Pharmacology, University of Copenhagen, Copenhagen, Denmark; 4 Research Unit for General Practice, Aarhus, Denmark; 5 Department of Clinical Pharmacology, Aarhus University Hospital, Aarhus, Denmark; 6 Unit of Clinical Pharmacology, Aalborg University Hospital, Aalborg, Denmark

**Keywords:** Complex Intervention, Deprescriptions, Clinical Practice Change, Dementia, Nursing Homes, General Practitioners

## Abstract

**Introduction:**

First-line treatment for behavioral and psychiatric symptoms of dementia is non-pharmacological. Still, psychotropic medication is widely used, despite its limited effect and harmful side-effects. More than half of all nursing home residents with dementia receive antidepressants, even though deprescribing is safe and feasible. Interventions to promote deprescribing of antidepressants in nursing homes are few and complex. To optimize the deprescribing process through an intervention, transparency for the development of the intervention is needed. We aim to describe the steps in the development and tailoring of an intervention targeting GPs, nursing home staff, and relatives to enhance collaboration on reducing the use of antidepressants in institutionalized older persons with dementia in Denmark.

**Method:**

A step-wise process guided by the core elements in the Medical Research Council constituted the tailoring process. Five steps were included; 1) a literature search, 2) interviews with stakeholders, 3) drafting the intervention prototype, 4) professionals’ assessment of the intervention, and 5) refinement of the intervention. The steps were conducted from June 2020 to June 2022.

**Results:**

Based on the literature search, interviews with stakeholders, and professionals’ assessment of the intervention, four main themes were identified; 1) focusing on antidepressants, 2) importance of professional qualifications, 3) collaboration and communication, and 4) patient and relative involvement. They guided intervention development and refinement of the final intervention, which included 1) a case-based training course and 2) a dialog tool including a symptom assessment scale to be used in a structured consultation at the nursing home.

**Conclusion:**

This study presents a detailed account of the tailoring process for a complex intervention to optimize deprescribing of antidepressants for older persons with dementia at nursing homes. By presenting a thorough development process, we expect to achieve increased adherence to the intervention which is currently being tested in an ongoing cluster randomized controlled trial. The transparency of the process will also increase the future development of other similar complex interventions.

**Supplementary Information:**

The online version contains supplementary material available at 10.1186/s12913-022-08961-9.

## Introduction

Behavioral and psychiatric symptoms of dementia (BPSD) include amongst others agitation, aggression, delusions, hallucinations, paranoia, wandering, depression, apathy, sleep disturbances, and a variety of behavioral disturbances [1, 2]. It is widely acknowledged that the first line of treatment for these symptoms is non-pharmacological strategies and a person-centered approach [3–6]. However, pharmacological treatment with antipsychotics and antidepressants is commonly used despite their limited effect and often harmful side effects [7–12]. Recent Danish guidelines for dementia recommend against the use of selective serotonin reuptake inhibitors (SSRIs) for BPSD and warrant caution when using other types of antidepressants because of their high anticholinergic burden [13, 14]. Reduction or deprescription of these types of medication can be performed safely; yet, their use among older persons with dementia living in nursing homes remain high [15–19]. A Danish study demonstrated that more than half of the residents receive antidepressants when they move into a nursing home and that the prescription of these drugs continues for at least a year after moving in [9].

Different interventions promote the appropriate use of psychotropic medication. The majority of these interventions focus on antipsychotics, while only a few have addressed the use of antidepressants. These interventions often focus solely on medication reviews conducted by a single health professional employing a deprescribing guideline (e.g. Beers criteria, STOP/START) [20, 21]. Although using such guidelines may prompt immediate changes in the patients´ medical chart, these studies do not explore if there is a sustainable change in the behavior of the prescriber nor what role the nursing home staff plays [22–26]. Furthermore, they do not take into account the complexity of a nursing home context [27]. Since many older persons with dementia live in nursing homes, any change in medication is part of a complex process involving at least a physician, nursing home staff, and relatives of the person with dementia. More recently, this has been acknowledged by several initiatives that focus on multidisciplinary teams to conduct medication reviews that include both nursing home staff, physicians, and pharmacists in the process [28, 29]. However, the lack of physician and staff qualifications as well as knowledge concerning deprescribing have proven to be important barriers [30]. While some studies have attempted to address these issues with success, a recent study using person-centered care to decline the use of antipsychotic medication showed no benefit, which suggests that there remains a need for further improvement [31–33].

Another important barrier to deprescribing is poor communication between physicians, nursing home staff, and relatives, however only a few interventions have focused on how to improve communication [18]. These interventions were based solely on literature research or on a development process that was poorly described. This makes it difficult to improve an intervention or disentangle why an intervention was unsuccessful [34]. For interventions to be effective, they depend on the coordinated efforts of the health professionals, the patient, and relatives [19, 35, 36]. Additionally, they often require several interacting educational and behavioral components in order to change the clinical behavior of the health professionals (e.g. general practitioners (GPs) or nursing home staff) [32]. Such interventions are considered complex because of the number of components in the intervention, the number of behaviors targeted, or the various expertise and skills demanded from the staff, which delivers the intervention [37, 38]. It has been recommended to adopt phased approaches when developing complex interventions, e.g. as outlined by the Medical Research Council´s (MRC) framework for developing complex interventions [37–41]. Such phases involve development, feasibility/piloting, evaluation, and implementation. They may be followed linearly or, if necessary, revisiting prior phases.

Our study is part of a larger project aiming to develop a robust model for the deprescribing of inappropriate antidepressants in nursing home residents with neuropsychiatric symptoms [42]. Using a phased approach, we aimed to develop a complex intervention to support the communication between GPs, nursing home staff, relatives, and patients in the process of deprescribing. In this paper we describe the steps of our tailoring process to ensure transparency in the development, which will allow others to replicate and gain insights into the learning process of developing complex interventions. Additionally, detailing the development process will add to the understanding of later trial outcomes, as the intervention will be tested in a cluster randomized controlled trial (cRCT) [42].

## Methods

### Setting

In Denmark, nursing homes can be either private or municipality-owned [43]. About half of the residents in a regular nursing home have a diagnosis of dementia or other causes of cognitive deficit [44]. Few nursing homes are intended only for residents with dementia. The staff at the nursing homes includes nurses, healthcare assistants, healthcare helpers, and other healthcare professionals. There is typically a high turnover of staff and the size of the staff group is variable. The educational level of the staff also varies greatly between nursing homes [45]. The majority of nursing homes have a GP that functions as a nursing home physician for some or all of the residents at the nursing home. The GP working as a nursing home physician typically attend about 20–40 residents at a nursing home. Most GPs have two to four days scheduled at the nursing home each month, where they follow up on their patients and conduct home visits. In addition to these scheduled days, the nursing home staff can contact the GP by phone or via e-mail consultation concerning the residents that the GP attends to. Nursing home residents may choose to keep their usual GP when they relocate to a nursing home. Consequently, the staff at a nursing home often has to refer to several GPs.

### Development of the intervention

The process of developing the intervention followed a step-wise approach inspired by the development phase of the MRC framework [38]. Based on the core elements of the MRC, in particular considering context, engaging stakeholders, identifying key uncertainties, and refining the intervention, our development process included five steps; 1) a literature search, 2) interviews with stakeholders, 3) drafting the intervention prototype, 4) professionals’ assessment of the intervention, and 5) refinement of the intervention (Table 1). To enhance transparency, methods and results of the qualitative sub studies were reported according to the COREQ-Checklist and the TIDier checklist was used for step 5 [46, 47].

**Table 1:** Overview of the steps in the development process

Steps	Name of the step	What was conducted	Date
Step 1	Literature search	• Qualitative systematic review	June—December 2020
Step 2	Interviews with stakeholders	• 1 GP • 2 Experts • 3 Patient/relative dyads	November 2020—February 2021
Step 3	Drafting the intervention prototype	• Dialog tool • Symptom assessment scale	February—April 2021
Step 4	Professionals' assessment of the intervention	Interviews with: • 4 GPs • 3 Nursing home staff groups	April—May 2021
Step 5	Refinement of the intervention	• Adjusting the intervention based on the interviews • Evaluation of the intervention with 1 expert (from step 2)	May—June 2022

The research team involved in the development included a female medical doctor (PH), a psychiatrist and epidemiologist (MPR), a GP (AH), and an implementation scientist with expertise in cross-sectional studies (GO).

### Step 1: literature search

First, we performed a literature review to gain insights into the barriers and facilitators of deprescribing psychotropic medication in a nursing home setting. It was carried out between June and December 2020. The search method was previously described [48].

### Step 2: interview with stakeholders

In step 2, we conducted qualitative, semistructured interviews with the primary stakeholders [49]. All the interviews were audio recorded and short summaries were made after each interview. The interviews included 1) two experts, one pharmacist working within the field of clinical pharmacology and pharmacy whom has conducted several deprescribing interventions in nursing homes prior, and one GP with a special interest in geriatrics and nursing homes, 2) three dyads of patients and relatives, and 3) one GP working as nursing home physicians. All participants represented a convenience sample; experts and GPs were approached by the research team’s network, and patient/relative dyads were invited through the general practice where a member of the research team worked (AH). The dyads were invited to participate by telephone, four accepted of whom one withdrew from the study. All participants signed informed consent before the interview. The patients were one female and two males between the ages of 70–75 with their spouses. All the patients had a diagnosis of dementia and none had been diagnosed with depression. One patient was receiving antidepressant medication.

The interviews with the GPs and patients/relatives followed a semi-structured interview guide and provided insight into the patients’ lives and the GPs’ and nursing home staff’s working routines which enabled us to plan and develop the intervention to be both user-friendly and clinically relevant. The interview guide was developed based on our findings from the qualitative systematic review and experience within the research team, especially GO who is an implementation scientist with expertise in cross-sectional studies. PH, a medical doctor, with experience in conducting medical interviews, conducted the interviews. All interviews lasted one to one and a half hours. The interviews with the GP and experts were conducted online, while the interviews with the patients/relatives were held at their homes.

The GPs were instructed to describe the experience with deprescribing psychotropic medication for patients with dementia living in nursing homes and the difficulties they had encountered. Based on the systematic review, the GPs were asked to differentiate between different classes of psychotropic medication (e.g. antipsychotics, antidepressants, hypnotics) and which problems they encountered for each class. In addition, questions about collaboration with the nursing home staff, patients, and relatives were included.

The interviews with the dyads of patients/relatives included questions to obtain knowledge about their lives and how they live with a diagnosis of dementia. Furthermore, they shared their perspective on medication with a focus on antidepressants and on what was important for them, when changing of medication was required. Moreover, they were asked specifically about what they would expect and require in a project like the one we planned to conduct.

Finally, interviews with experts within the field of clinical pharmacology and pharmacy and a GP with a special interest in geriatrics and nursing homes were conducted. They constituted open-ended questions to gain additional knowledge into the Danish setting concerning the working conditions of a nursing home physician and how they work with older persons with dementia, medication, and deprescribing. In addition, questions on how prior interventions for this population had been conducted and which pitfalls to avoid were included.

The interviews were transcribed verbatim and analyzed by the first author PH following the steps of Braun and Clark’s thematic analysis [50]. Final analysis was agreed on between first and last author (PH and GO).

### Step 3: drafting the intervention prototype

We designed and developed the intervention in a step-wise process, taking into account the insights gained in steps 1 and 2. In the intervention prototype, we wanted to include two primary elements: 1) a dialog tool and 2) a symptom assessment scale. Therefore, we searched PubMed to identify existing dialog tools specific to nursing home residents with dementia and GPs. The following terms were used in different combinations: “Dementia”, “Deprescription”, “Health Communication”, “Interprofessional Relation”, “Dialog Tool” and “Communication Tool”. Furthermore, we searched for symptom assessment scales for patients with dementia and how they were used in the Danish nursing homes to ensure the patients’ symptoms were addressed. This led to an initial draft of the dialog tool combined with the symptom assessment scale, which was designed and printed for assessment by the GPs and nursing home staff in step 4.

### Step 4: professionals’ assessment of the intervention

To assess the intervention, we conducted interviews with four different GPs and three groups of nursing home staff. We chose this approach, as the intervention was still at a preliminary stage and it would be too early to conduct an actual pilot study.

A convenience sample of GPs and nursing home staff were recruited through the research team’s network. All GPs and nursing home staff were from a region located outside the future RCT’s region and would, therefore not be eligible for participation in the RCT. These semi-structured interviews were carried out at the GPs office or in the nursing home. They lasted approximately one hour. We conducted individual interviews with GPs and group interviews with nursing home staff with 6–10 participants. The nursing home staff included nurses, healthcare assistants, healthcare helpers, and management staff. The GPs were interviewed individually since they often work alone at the nursing homes. The nursing home staff work as a team and it was therefore important to conduct the interviews as a group to get the inputs from as many participants as possible. Furthermore, interviews were also conducted as either individual or group, according to what was practically possible.

GPs and nursing home staff were inquired about their daily work life, their collaboration, and their experiences with patients/residents with dementia. Furthermore, they were specifically asked about medication and their experience with deprescribing of antidepressants and other psychotropic medication.

The background for the intervention was explained in detail to them. Subsequently, they should comment on whether GPs and nursing home staff found the intervention relevant and if they could imagine using the intervention.

For the GPs, the focus was on both the meeting in general, the dialog tool, and the symptom assessment scale, since the dialog tool and the symptom assessment scale would be their instrument to structure the meeting at the nursing home with a focus on deprescribing of antidepressants. For the nursing home staff, the interview focused on the symptom assessment scale and how to use it. Furthermore, we explored their thoughts concerning the meeting at the nursing home with the GP. They were all encouraged to remark upon potential barriers or come up with ideas on how to improve the meeting. Finally, the prototype intervention was shown to them, and they were asked to think through the method of the intervention, using one of their patients that they know well as an example. For the GPs, this included both the dialog tool and the symptom assessment scale. The nursing home staff were asked to fill out the symptom assessment scale and comment on its usability. Both groups were also asked to comment on the layout.

These interviews were also transcribed verbatim and analyzed by the first author PH following the steps of Braun and Clark’s thematic analysis [50]. Final analysis was agreed on between first and last author (PH and GO).

### Step 5: refining the intervention

Based on the views expressed by the GPs and nursing home staff during the interviews in step 4, a final refinement of the intervention was performed. The intervention was then discussed with the expert GP with a special interest in geriatrics and nursing homes.

## Results

Based on the insights gained in step 1, 2, and 4 (Table 1), we developed and tailored a complex deprescribing intervention. During these first three steps, four main themes were identified: 1) focusing on antidepressants, 2) importance of professional qualifications, 3) collaboration and communication, and 4) patient and relative involvement. These themes informed the final version of the intervention and will be elaborated in the following sections.

### Focusing on antidepressants

From the systematic review, we identified five main factors that promoted and inhibited deprescribing psychotropic medication for institutionalized older persons with dementia. These concerned 1) operationality and routines; 2) lack of resources and qualifications; 3) patient-related outcomes; 4) policies; and 5) collaboration [48]. Additionally, we identified very few studies with a specific focus on antidepressants, despite the high amount of usage in the population. This led us to incorporate questions in the interview guide concerning stakeholders’ experience with antidepressants usage as well as barriers and facilitators for deprescribing antidepressants to further explore the importance of this theme.

### Importance of professional qualifications

Based on the systematic review and the interviews with stakeholders, the importance of professional qualification became clear. GPs found that psychotropic medication initiated by another specialist (e.g. a psychiatrist) was difficult to deprescribe without consulting a specialist in psychiatry. This was due to fear of worsening symptoms in the patient and lack of confidence in their own skills as well as resistance from the nursing home staff.

*"It takes quite an effort [treatment of behavioural symptoms and dementia]. It’s hard for the staff and it’s hard for me. It seems to me that we often get the gerontopsychiatrists to treat the most difficult cases…but I think we could* *do a lot of it by ourselves if we were properly equipped for it.” (GP no. 3) *However, if deprescribing of antidepressants was the focus, the GPs were more inclined to deprescribe, even if the treatment had been initiated by other specialties. Both experts and GPs addressed the need for tools to deprescribe antidepressants, since it was seen as difficult to know when to stop the medication. The lack of non-pharmalogical alternatives were also emphasized as a barrier. In addition, some GPs expressed concerns about the educational level of the nursing home staff.

Nursing home staff also expressed concern about deprescribing psychotropic medication, especially antipsychotic medication. They were aware that the use of antipsychotic medication should be diminished but felt more comfortable when a gerontopsychiatrist was involved. The nursing home staff did not believe the GP had the specialist training to deprescribe antipsychotic medication. However, many had experienced successful attempts with GP-initiated deprescribing of antidepressants. A healthcare assistant explained:

*“We had someone [a patient who received antidepressant medication]. It worked out fine. But she [the patient] wasn’t happy that we did it. But we said, let’s try it. We don’t tend to think that it has much of an effect [the* *medication]. It was* *fine. There was no problem whatsoever. (Healthcare assistant, nursing home no. 1) *The interviewer identified insufficient qualification both among nursing home staff and GPs as a barrier to deprescribing. Such concern was not as prominent when the focus was narrowed down from all psychotropic medication to antidepressants.

The GPs did, however, express some concerns about the quality of the observations from nursing home staff, if they did not know the staff well enough, which occurs often due to the high turnover in staff at nursing homes and the staff working in shifts (day, evening, night).

### Collaboration and communication

Collaboration and communication were found to be important for all attempts to deprescribe. Our systematic review identified that collaboration and communication could be both facilitating and impeding.

The interviewed experts (GP with a special interest in geriatrics and nursing homes and physician in clinical pharmacology) and GPs both emphasized the need to have a qualified working collaboration with the nursing home staff and, to a certain extent, also the relatives. They highlighted planned GP-visits at the nursing home several times per month and trust between nursing home staff and GP as important factors for a good working collaboration. The GPs reported relying on the observations the staff supplied to decide when to attempt deprescribing, while also acknowledging that sometimes they were unsure if the nursing home staff knew, which information was relevant to convey.

Nursing home staff reported that it was easier to make changes, when they felt heard by the GP and had a good relationship with the GP. However, the nursing home staff sometimes felt that the GP wished to make changes too fast and that they as nursing home staff were more inclined to be cautious.

*"You could say that we [nursing home staff] are probably a bit more cautious, because if the residents appear to us to be in a stable phase, then we may not think it necessary to tamper too much with the medication. As long as* *they [the residents] do not appear to be lethargic or otherwise affected, but are in a stable phase, then we may not see any reason for tampering with the medication” (nurse, nursing home no. 1) *Both nursing home staff, experts, and GPs suggested that to ensure relevant information, improve communication, and avoid unnecessary information to the GP, a clear structure for communicating with one another was needed. This could be achieved in different ways, but it should include a clear understanding from both parts of what to expect when deprescribing and which symptoms the nursing home staff needed to be observant of as well as how to handle uncertainties. One of the experts, the clinical pharmacist with experience in deprescribing studies in Danish nursing homes, suggested using the Neuropsychiatric Inventory – nursing home version (NPI-NH) [26, 51]. The symptom assessment scale was considered by both nursing home staff and GPs as a means to ensure a common language concerning what was relevant to report from nursing home staff to the GP. The symptom assessment scale was found useful both to prompt further discussion about the present symptoms and to enquire information about the workload experienced by the nursing home staff due to the symptoms.

### Patient and relative involvement

In the interviews with stakeholders, it became apparent that the theme concerning patient and relative involvement was important. Relatives found it difficult to think about changes in the medication regime but agreed that if the physician deemed it relevant to deprescribe, they would agree to this. The relatives did perceive it as relevant to be engaged in the adjustments of the patient’s medication. One relative even deemed it irrelevant that the patient should be present at the meeting with the GP:

*"Well, I do not think it’s entirely relevant for him [the patient] to participate [at a structured meeting at the nursing home]. Because it, it.. well, I mean, he wouldn’t get anything out of it, and neither would you. He might well say yes, we can do it, but he wouldn’t get anything out of it. But I do believe that we, the relatives, well at any rate I would, like to hear the arguments for doing it [deprescribing] and what could happen if it were attempted. I* should think *that would be very relevant.” (Relative no. 2).*

GPs and nursing home staff expressed hesitation concerning the involvement of relatives and patients. In their experience, the patients at the nursing homes could not participate in a dialog as presented in the intervention in a meaningful way. Furthermore, both nursing home staff and GPs were unsure how much extra and relevant information a relative could add. Moreover, they believed that many relatives did not want to be involved and those who did, often were a bit “too” involved. The GPs found it very time-consuming if they should contact relatives prior to the meeting at the nursing home. Both GPs and nursing home staff pointed out that it was more efficient if the staff at the nursing home contacted the relatives. A GP expressed it this way,

*"The thing about including the relatives. I don’t know quite what I think about that. I mean, the relatives, they have very different approaches to… Some relatives are very, well not actual authoritative, but protective of their father or mother or mother-in-law or whatever the relationship might be. As if they [the relatives] know best, because they know the person. And they can be difficult to deal with in such situations.” (GP no. 4) *Thus, both GPs and nursing home staff found the intervention more feasible, if it was not mandatory to include relatives in the meeting. GPs and nursing home staff stated that the relatives could have relevant information concerning the patients and that the relatives' views should be included. However, they were concerned about the personal aspect of the relatives concerning the patients and that this could lead to some difficult situations, which would not be relevant for the meeting. A nurse explained,

*"There’s also the thing about the personal relationship and also the professional relationship. Of course, we have a different perspective on their loved ones than they do. And obviously there’s some history there. And again, some* *of those things may undeniably be very worthwhile knowing about: but there is constantly the thing about us looking at things from a professional perspective and them looking at things from a personal perspective. So, if it* *were possible to coordinate all this [relatives’ perspective included] then the communication should be through us [nurses] to get the best result” (nurse, nursing home no. 1) *GPs supported that the nursing home staff who knew the relatives well had the primary contact with the relatives. Furthermore, both GPs and nursing home staff deemed it most relevant that the nursing home staff decided on when to include the relatives.

All stakeholders suggested that medication changes should not be attempted until 3–6 months after the patient had moved to the nursing home, to help the patient adjust to the new setting. This also ensured that the nursing home staff was better acquainted with the patient and their relatives and could provide better information on how and when to involve both the patient and the relatives.

### Drafting the intervention

The prototype intervention was drafted during step 3 in the development process. It was informed by the preliminary findings in the two prior steps (step 1; literature search and step 2; interview with stakeholders) and included two elements; a dialog tool and a symptom assessment scale.

From the systematic review and the interviews with stakeholders, it became clear that there was a need for narrowing down the focus of the intervention from all psychotropic medication to antidepressants.

Based on the main findings concerning collaboration and communication, the dialog tool was intended to be used at a structured GP consultation at the nursing home. Our search for existing dialog tools in step 3 yielded no relevant articles. Therefore, to tailor the dialog tool in a way that Danish GPs could use and to optimize communication and collaboration, we applied a consultation process as a template that provided a structure to the professional communication [52]. This consultation process is widely used by Danish GPs and is based on the Cambridge-Calgary Observation guide [53]. It includes a structure for the consultation to ensure that the patient's thoughts, concerns, and plans are taken into consideration, thereby building a shared agenda between the GP and the patient throughout the consultation.

To further incorporate collaboration, communication, and professional qualifications, the Neuropsychiatric Inventory – nursing home version (NPI-NH) was used as inspiration to construct the questions in the dialog tool [51]. The NPI-NH addresses the symptoms of patients with dementia at nursing homes from the staffs’ perspective and a simplified version of the NPI-NH questionnaire was included as part of the dialog tool as well.

To support the involvement of relatives/patients in the deprescribing process, the dialog tool included questions addressing the patients and relatives’ view on medication. Furthermore, GPs and nursing home staff were reminded to invite them to the meeting.

The views of the GPs and nursing home staff in step 4 further unfolded the four main themes and helped refine the intervention. The NPI-NH was included as a symptom assessment scale to support the nursing home staff in observing the patient and communicating with the GP about the patient's symptoms. To achieve increased focus on professional qualification, a case-based training course was constructed and incorporated as part of the intervention. The course included basic knowledge concerning dementia, alternatives to medication, why medication is not always the right choice, and what symptoms to look for when tampering with medication. The course was constructed as a slideshow with fictive patient cases to prompt discussions between GPs and nursing home staff. It was delivered and taught by the GPs to the nursing home staff before initiating the meetings concerning deprescribing at the nursing home. The training course was added to the intervention to increase knowledge for both GPs and nursing home staff and to ensure a shared starting point before initiating the meetings.

Overall, the GPs were satisfied with the dialog tool and the symptom assessment scale. They found the intervention relevant, it addressed the situations they often found themselves in, and it was found easy to use. The nursing home staff also agreed that the intervention was relevant and usable. The symptom assessment scale was known to many at the nursing home and made sense for them to use.

### Refinement of the intervention

In step 5, important refinements concerning the involvement of patient/relatives were made to the intervention. Even though the relatives stressed the relevance of being included, both GPs and nursing home staff were more hesitant. They had concerns about the time aspect of including the relatives and that some of the personal views of the relatives would not be fruitful for the meeting. To minimize the risk of the GPs not using the intervention due to resistance towards including relatives, the involvement of relatives was adjusted to be optional.

Thus, the final intervention was a structured consultation between the GP, nursing home staff and when deemed relevant, the relatives and patients at the nursing home. The focus of the consultation was through communication and involvement, to attempt reduction or deprescribing of antidepressant medication. Prior to the structured meeting, all GPs had to conduct a case-based training course to instruct the nursing home staff. The training course consisted of a slideshow with information on person-centered care, the use of antidepressants and what to be aware of when reduction of antidepressant medication was attempted. The structured consultation should be guided by the developed dialog tool which included 1) guidance questions for the GP to initiate conversation and evaluate the concerns of the nursing home staff and 2) a symptom assessment scale based on the NPI-NH to further explore the symptoms of the patients. See TiDieR checklist in additional files (Additional File 2).

A detailed description of how the intervention is to be used can be found in our protocol for the RCT [42].

## Discussion

This paper describes the development and tailoring process for a complex intervention aiming to support communication between GPs, nursing home staff, relatives and patients with a view to deprescribe antidepressants in institutionalized older persons with dementia. Four themes were identified in the qualitative interviews: 1) focusing on antidepressants, 2) importance of professional qualifications, 3) collaboration and communication, and 4) patient and relative involvement. These themes guided the tailoring process, which resulted in a two-component intervention; 1) a case-based training course and 2) a structured consultation at the nursing home using the developed dialog tool including a symptom assessment scale.

The nature of the problem concerning deprescribing in older persons with dementia is complex and, therefore, requires complex interventions in order to succeed [54]. The MRC framework for complex interventions provides a structure increasing the chance that professionals understand and agree with the new practice [38].

Case-based training and other sessions to improve knowledge and enhance a deprescribing culture has proved to be somewhat efficient in deprescribing but can be time consuming [32]. Structured consultation e.g. by means of checklists such as our dialog tool have additionally shown to be efficient to secure that deprescribing is actually put on the agenda, however it does not necessarily last for a longer period [55, 56].

Furthermore, it is essential that the new practice is realistic and relevant in the professional’s clinical setting [57]. To increase the success of this, the development phase of a study is essential. Other studies concerning deprescribing have focused on the development phase as well, using theories, including the MRC [34, 58]. Including stakeholders throughout the process enabled us to identify four central themes and take into account the key challenges they posed.

The importance of patient and relative involvement in interventions has been stressed and incorporated in other studies [59–62]. Relative and patient involvement is increasingly important in research as well as in clinical decision-making, and dilemmas arise on how involvement is best achieved. In our study, we found that even though relatives believed it was important to be included, both the GPs and the nursing home staff saw it as a challenge in the intervention. This constitutes a major dilemma with involvement, because even if the professionals are skeptical concerning the importance of information from the relatives, the relatives may be more inclined to participate if they felt listened to. This would be an important topic to further investigate in another study. The themes concerning collaboration and communication as well as the importance of professional qualification were also found to be both facilitating and impeding in the systematic review [48]. These findings were supported and further elaborated in our interviews. Professional qualification as a barrier could be seen as an actual lack of qualifications but may also represent a lack of professional trust and knowledge about competencies between different health professionals. In line with previous research, insufficient time to make changes to the medication and a general view about not changing what is currently working were also found to be significant barriers to the deprescribing process [63, 64]. Still, our development work showed that although some barriers persist to deprescribing of antidepressants, the GPs are more forthcoming in attempting to deprescribe antidepressants over antipsychotics, which have also been shown to be feasible in other studies [63, 65, 66]. The reason for this is not fully elaborated in our study, but a hypothesis is, that the GPs are not as worried about withdrawal symptoms and that they generally do not believe there is an actual effect of the antidepressant medication.

### Strengths and limitations

One of the strengths of this study is the detail and transparency with which we present it. Only a few articles are published on the pre-pilot testing, explaining how an intervention was developed. This makes it easier for others to reproduce and use what is relevant in their setting. Furthermore, we had several stakeholders involved early in the process. This strengthened our intervention, because views from central stakeholders were taken into consideration. However, we do acknowledge that the patient and relative perspectives could be improved since they were only included at the beginning of the process. Additional none of the patients were residents in a nursing home and only one patient were currently receiving antidepressant medication. Furthermore nursing home staff was not included as part of the early stakeholders, which is a limitation. Their insights into how they are working with patients, relatives and GPs could have provided valuable insights that would have improved the intervention that was presented to the nursing home staff later on.

All the interviews were conducted by a medical doctor with primary experience within the medical interview technique. We acknowledge that conducting a qualified interview is based on both a thorough interview guide and a qualified interviewer. Since our interviewer had only limited experience, this may constitute a limitation. However, the interview guides used were developed in cooperation with an implementation science specialist to ensure the quality of the interviews was upheld.

Several steps of the development process were complicated by the COVID-19 pandemic. Firstly, no nursing home staff was included in the early process. Secondly, it was not possible to include patients living in nursing homes, since all non-essential visitation to the nursing homes was banned. Thirdly, it was impossible to join a GP at the nursing home one day to get insights into how the work routines function and to interview nursing home staff as a primary stakeholder. Fourthly, it was not possible to schedule a follow-up meeting with the interviewed patients and relatives. Fifthly, we had planned a pilot study to be conducted in continuation of the development in line with the MRC before initiating the evaluation of the intervention, which was not feasible.

All the limitations that occurred due to the COVID-19 pandemic have hindered some important insights. The follow-up meetings with the patients could have provided additional insight from the patient/relative perspective concerning the reluctance of GPs and nursing home staff to include them in the process. Research has also shown that people with dementia may be able to participate more actively than we earlier anticipated [67] and acquiring insight from the population the intervention is planned to address would have been a major improvement. The limitation of lack of involvement of nursing home staff in the early phase and not being able to visit a nursing home with the GP have been somewhat diminished by our conduction of several interviews with different professionals and stakeholders, which provided a broad perspective into the working routines at nursing homes.

This may have yielded important insights that did not come forth in the development process such as how the health professionals used the intervention in practice and how patients and relatives were involved. A process evaluation will take place during the trial to obtain a better understanding of what is working well and what could be improved.

## Conclusion

Our results from this study add to the growing body of research on how to practically develop an intervention to deprescribe. Furthermore, our study stresses the importance of comprehensive developmental work for multi-component interventions to succeed in changing clinical practice and provides transparency to the complex detangling of mechanisms that affect deprescribing in clinical practice. Currently, the trial evaluating the intervention is being carried out, and the insights from this development study will add to the interpretation of trial outcomes. With time, we hope this will help to emphasize the integration of deprescribing as a natural part of the medical prescribing culture.

## Supplementary Information

**Additional file 1. **COREQ COnsolidated criteria for REporting Qualitative research) Checklist.

**Additional file 2. **TiDieR checklist for interventions.

## Data Availability

The research team has full access to all data. The data including the interview guides that support the findings of this study are available on request from the corresponding author PH. The data are not publicly available due to them containing information that could compromise research participant privacy. (copied from: https://www.springernature.com/gp/authors/research-data-policy/data-availability-statements/12330880).

## Abbreviations

GP: General practitioner
MRC: Medical research council
NPI-NH: Neuropsychiatric inventory, nursing home
BPSD: Behavioral and psychiatric symptoms of dementia
SSRI: Selective serotonin reuptake inhibitors
cRCT: Cluster randomized controlled trial
